# T Cells Control Chemokine Secretion by Keratinocytes

**DOI:** 10.3389/fimmu.2019.01917

**Published:** 2019-08-09

**Authors:** Tabea Rauschenberger, Viola Schmitt, Muhammad Azeem, Stefan Klein-Hessling, Krisna Murti, Franziska Grän, Matthias Goebeler, Andreas Kerstan, Matthias Klein, Tobias Bopp, Edgar Serfling, Khalid Muhammad

**Affiliations:** ^1^Department of Molecular Pathology, Institute of Pathology, University of Würzburg, Würzburg, Germany; ^2^Comprehensive Cancer Center Mainfranken, Würzburg, Germany; ^3^Department of Dermatology, Venereology and Allergology, University Hospital Würzburg, Würzburg, Germany; ^4^Institute for Immunology, University Medical Center, University of Mainz, Mainz, Germany; ^5^Research Center for Immunotherapy (FZI), University Medical Center, University of Mainz, Mainz, Germany; ^6^Department of Immunology, University Cancer Center Mainz, University Medical Center, University of Mainz, Mainz, Germany

**Keywords:** chemokine, keratinocytes, IFN, lichen planus, T cells, Nfatc1

## Abstract

The massive infiltration of lymphocytes into the skin is a hallmark of numerous human skin disorders. By co-culturing murine keratinocytes with splenic T cells we demonstrate here that T cells affect and control the synthesis and secretion of chemokines by keratinocytes. While pre-activated CD8^+^T cells induce the synthesis of CXCL9 and CXCL10 in keratinocytes and keep in check the synthesis of CXCL1, CXCL5, and CCL20, keratinocytes dampen the synthesis of CCL3 and CCL4 in pre-activated CD8^+^T cells. One key molecule is IFN-γ that is synthesized by CD8^+^T cells under the control of NFATc1 and NFATc2. CD8^+^T cells deficient for both NFAT factors are unable to induce CXCL9 and CXCL10 expression. In addition, CD8^+^T cells induced numerous type I IFN-inducible “defense genes” in keratinocytes encoding the PD1 and CD40 ligands, TNF-α and caspase-1. The enhanced expression of type I IFN-inducible genes resembles the gene expression pattern at the dermal/epidermal interface in lichen planus, an inflammatory T lymphocyte-driven skin disease, in which we detected the expression of CXCL10 in keratinocytes in close vicinity to the infiltration front of T cells. These data reflect the multifaceted interplay of lymphocytes with keratinocytes at the molecular level.

## Introduction

Interactions between keratinocytes and leukocytes play a decisive role in the development of numerous skin diseases. Chemotactic chemokines attract leukocytes to the skin and direct them to the sites of inflammation, infections, and wounds (1, 2). Keratinocytes synthesize a restricted set of chemokines whose synthesis is amplified by numerous stimuli, including type I IFNs, IFN-γ, TNF-α, and others that, to a large part, are produced by lymphocytes and other cells. In addition, the infiltration of lymphocytes into the skin induces a pattern of chemokines that are either synthesized at very low amounts or not at all by keratinocytes under homeostatic conditions (3). Although there have been numerous attempts to elucidate the role of individual chemokines in the generation of skin diseases, our current knowledge on the interplay of the immune system with the skin and the function of the chemokine network in skin physiology and pathology is far from clear.

There are ~50 chemokines and 20 chemokine receptors (4) that, in addition to control leukocyte traffic (5), play an important role in the differentiation and function of lymphocytes during immune reactions. Both in bone marrow and thymus the development of lymphoid cells is guided by the interaction of chemokines with their receptors (6, 7). In periphery, due to the expression of chemokine receptor CCR7, memory T cells have been classified into CCR7^+^ central memory T_CM_ cells that are enriched in lymph nodes and tonsils, and CCR7^−^ effector memory T_EM_ cells that are enriched in non-lymphoid organs (8). Several chemokine receptors including CCR4, CCR8, and CCR10 have been described to direct T cells to the inflamed skin and orchestrate T cell-keratinocyte interactions (9, 10).

The differentiation of keratinocytes in the epidermis, the first barrier of the organism against the environment, is a fine-tuned process that is disturbed under pathological conditions. Keratinocytes of the *stratum basale* proliferate, detach and migrate to the *stratum spinosum* and *stratum granulosum* and finally differentiate to corneocytes of the *stratum corneum*. Under physiological conditions this process takes 1 week in mice and 4 weeks in humans (11). The cells of the *stratum basale* are in close contact with the basement membrane and the dermis in which the majority of skin lymphocytes patrol. In skin disorders there is often a massive infiltration of lymphocytes into the dermis and, less frequently, into the epidermis. Infiltrating lymphocytes affect the proliferation of keratinocytes and disturb their differentiation to corneocytes. Lichenoid dermatoses such as cutaneous lupus erythematosus and lichen planus (12) are prototypic skin disorders that are characterized by a massive infiltration of cytotoxic lymphocytes and the “liquefaction degeneration” of the basal layer of the epidermis. Those infiltrating lymphocytes show a high expression of the chemokine receptor CXCR3, the common receptor of the chemokines CXCL9, CXCL10, and CXCL11. Due to their massive IFN-γ production, infiltrating T cells induce the synthesis of CXCL9 and CXCL10 in keratinocytes that, on the other hand, perpetuate and accelerate the further infiltration of activated T cells to the skin (13–15).

To elucidate the molecular events that orchestrate the interplay between keratinocytes and skin-infiltrating CD8^+^T cells we co-cultivated both types of cells and determined their chemokine profile. While similar to other authors (3) we detected a strong induction of the chemokines CXCL9 and CXCL10 in keratinocytes by IFN-γ, we observed that CCL3 and CCL4, which are expressed by cytotoxic T cells under the control of NFAT factors, are inhibited. On the other hand, CXCL1, CXCL5, and CCL20 expressed by keratinocytes are kept in check by T cell-derived IFN-γ. The massive induction of CXCL9 and CXCL10 is linked to the expression of NFATc1 and NFATc2 since CD8^+^T cells deficient for these NFATs were unable to stimulate both chemokines. In lichen planus patients who show a massive T cell infiltration into the skin we detected the expression of CXCL10 in keratinocytes near the invasion front. In addition to chemokines, the co-culture of CD8^+^T cells with keratinocytes resulted in the expression of a number of type I IFN-inducible “survival molecules” by keratinocytes such as the PD1 and CD40 ligands, TNF-α and caspase 1 that affect the fate of keratinocytes.

## Materials and Methods

### Mice, Isolation and Culture of Cells

Eight to twelve weeks old C57/B6, sex-, and age-matched mice were used in the experiments. Transgenic mice expressing NFATc1-bio protein as well as the *Nfatc1*^*flx*/*flx*^ *x Cd4-cre* and *Nfatc2*^−/−^ mouse lines have been described previously (16). NFAT DKO mice were generated by crossing of *Nfatc1*^*flx*/*flx*^ *x Cd4-cre* and *Nfatc2*^−/−^ mice. Primary murine keratinocytes were either isolated from tails of adult mice or from newborn mice according to Lichti et al. (17) with slight modifications. Both mouse tails and newborn mice were disinfected using 7.5% povidone-iodine solution followed by extensive washes in 70% ethanol and distilled water. Upon preparation of skin, skin pieces were incubated overnight in dispase solution at 4°C followed by a short digestion in trypsin and incubation for 6 h in S-MEM (Gibco) medium containing 1.3 mM Ca^++^. After 6 h, cells were replenished with keratinocyte SFM medium containing 0.06 mM Ca^++^ (Gibco) for further culture. Since porcine trypsin kills murine keratinocytes, all cell culture divisions were performed with TrypsinLE (Gibco; # 12605-010). Keratinocyte cultures were treated with murine IL-1α (Preprotech 211-11A; 30 ng/ml), murine TNF-α (Preprotech 315-01A; 30 ng/ml), or murine IL-25 (Biolegend 587302; 2 μg/ml) for 1 and 3 d. To some cultures, neutralizing Abs directed against IFN-γ (Biolegend 505702; 5 μg/ml) or 100 ng/ml CsA were added.

All co-cultures of keratinocytes with CD8^+^T cells were performed at least 3 times in duplicate. Splenic T cells were isolated using mouse CD8 microbeads kit (Miltenyi Biotech, #130-049-401) according to the manufacturer's instructions and cultured in X-VIVO™ 15 medium (Lonza, BE02-060F). Cells were pre-activated by αCD3/CD28 (5 μg CD3ε, clone 73.51, BD Pharmingen) and CD28 (2 μg, clone 145-2C1, BD Pharmingen), diluted in 1 ml PBS for coating. The same number or 2-fold more pre-activated T cells were co-cultured for 1 or 3 d with freshly prepared keratinocytes. Due to the strong differentiation effect of Ca^++^-containing X-VIVO medium on primary keratinocytes, all co-cultures contained 4 parts SFM and 1 part X-VIVO medium.

### Transcriptome Analysis by Next Generation Sequencing (NGS)

Keratinocytes, either cultured alone, or after co-culture with CD8^+^ T cells, were deep-frozen in liquid nitrogen. Their RNA was extracted using Qiagen's RNeasy plus mini kit. The quantity of RNA was assessed using a Qubit 2.0 fluorometer, the quality of RNA was determined on a Bioanalyzer 2100 using a RNA 6000 nano chip. RNA-Seq libraries for NGS were prepared using 20 ng total RNA (RIN>8) and the NEBnext ultra II RNA Library Prep kit following the manufacturer's instructions. Barcoded RNA-Seq libraries were onboard clustered using HiSeq Rapid SR Cluster Kit v2 using 8 p. m. and 59 bps were sequenced on an Illumina HiSeq2500 using a HiSeq Rapid SBS kit v2. Sequencing raw data were further processed using the software CLC Genomics workbench (v7.5.1 with CLC's default settings for RNA-Seq analysis). Reads were aligned to the GRCm38 genome. For the pathway enrichment analysis (shown in Table 1 and Supplementary Table 1) the GORILLA (gene ontology enrichment analysis and visualization) tool was used (18).

**Table 1:** Compilation of the first top 10 pathways that were changed in murine keratinocytes upon co-culture with pre-activated CD8^+^ T cells for 1 d.

**GO term**	**Description**	***P*-value**	**FOR g-value**	**Enrichment (N, B, n, b)**
G0:0035456	Response to interferon-beta	1.05E-28	1.35E-24	47.44 (8048,39,87,20)
G0:0035458	Cellular response to interferon-beta	2.46E-28	1.59E-24	62.01 (8048,32,73,18)
G0:0051707	Response to other organism	1.55E-25	6.64E-22	8.37 (8048,271,149,42)
G0:0043207	Response to external biotic stimulus	3.63E-23	1.17E-19	6.68 (8048,364,149,45)
G0:0006952	Defense response	6.54E-23	1.68E-19	8.33 (8048,411,87,37)
G0:0009607	Response to biotic stimulus	2.70E-22	5.79E-19	6.38 (8048,381,149,45)
G0:0009617	Response to bacterium	1.19E-20	2.19E-17	4.82 (8048,141,604,51)
G0:0002376	Immune system process	1.28E-20	2.06E-17	4.45 (8048,703,144,56)
G0:0006955	Immune response	7.31E-19	1.05E-15	5.98 (8048,345,160,41)
G0:0051704	Multi-organism process	2.85E-18	3.67E-15	6.21 (8048,436,113,38)

*RNAs of keratinocytes were isolated and their transcriptome determined in NGS assays. Five hundred three genes whose expression was changed 2.5 fold between keratinocytes co-cultured with CD8^+^ T cells compared with keratinocytes alone were classified using the Gorilla (**G**ene **O**ntology en**RI**chment ana**L**ysis and visualization) tool (18). GO, gene ontology terms. FDR (False Discovery Rate) q-value is the correction of the indicated p-value for multiple testing using the Benjamini and Hochberg method. The enrichment (N,B, n,b) is defined as follows: N is the total number of genes, B is the total number of genes associated with a specific GO term, n is the number of genes in the top of the user's input list (or in the target set when appropriate), b is the number of genes in the intersection. Enrichment = (b/n)/(B/N). The individual genes in the 10 intersections are presented in Supplementary Table 1*.

The data of NGS assays have been deposited in the GEO database (GSE131905).

### Immunohistochemistry

All specimens were obtained from the archive of paraffin-embedded diagnostic tissues of the Department of Dermatology at the University Hospital of Würzburg, Germany, and were used in accordance to the guidelines of the local ethics committee. Anonymized sections were prepared from formalin-fixed, paraffin-embedded skin biopsies from lichen planus patients. Immunohistochemical stains were performed using mAb specific for CD8 (clone SP-16, dilution 1:50, Invitrogen), NFATc1 (clone 7A6, dilution 1:100, BD Pharmingen), NFATc2 (clone D43B1, dilution 1:200, Cell signaling), CXCL9 (MIG, dilution 1:15, R&D systems), or CXCL10 (Polyclonal, clone 33036, Dilution 1:15, R&D Systems) following the manufacturer's instructions. The stained slides were captured with confocal microscope (Zeiss) or a Nikon Ti motorized stage fluorescence microscope at the magnifications indicated. The absolute numbers of keratinocytes expressing CXCL10 were determined using ImageJ.

### Enzyme-Linked Immunosorbent Assay (ELISA)

The concentration of IFN-γ in the culture supernatants was determined using commercially available mouse IFN-γ ELISA kits (88-7314-88, Invitrogen). Each sample was analyzed in duplicate following the manufacturer's instructions.

### Chemokine Secretion Assay

For chemokine measurements, we used the LEGENDplex™ Mouse Proinflammatory Chemokine Panel (BioLegend) kit according to the manufacturer's instructions. This is a multiple fluorescence-encoded bead-based assay allowing the simultaneous detection of 13 different chemokines by flow cytometry.

### Statistical Analysis

Statistical analyses were performed using GraphPad (Prism) software, version 6.0. Data were presented as mean ± SEM. Unpaired *t*-test was performed to evaluate the statistical significance. Statistical significances are indicated (^***^p < 0.001, ^**^p < 0.005, and ^*^p < 0.05).

## Results

### Secretion of Chemokines by Primary Murine Keratinocytes *ex vivo*

**Figure 1:**
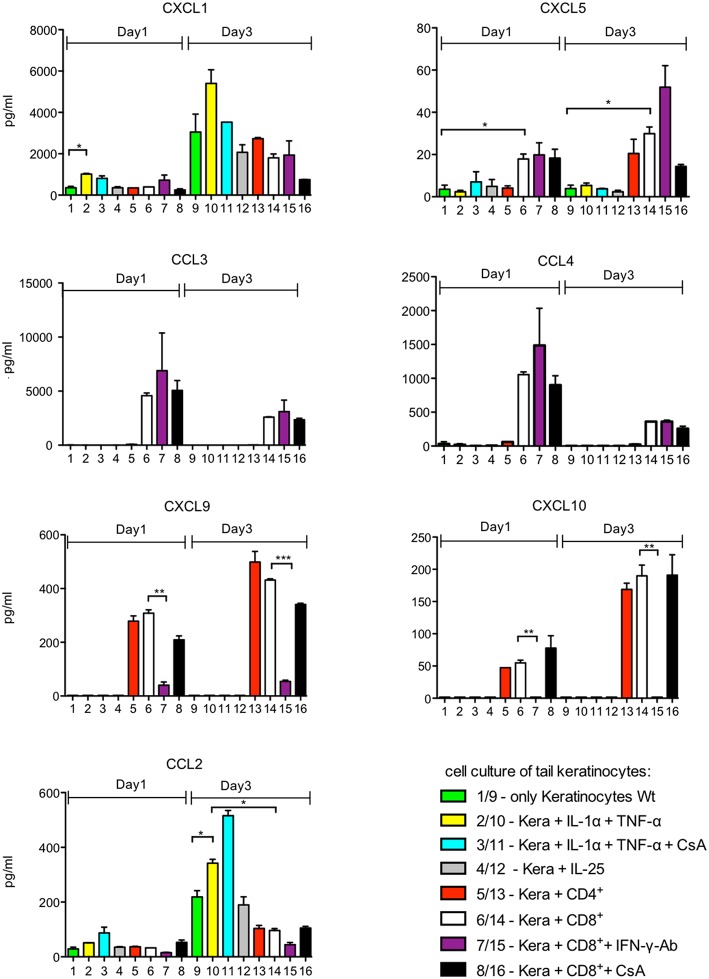
Chemokine secretion of murine keratinocytes *ex vivo*. 10^5^ keratinocytes cultured for 7 d *in vitro* were seeded in 12-well plates. They were cultivated alone (columns 1–4 and 9–12) or together with 10^5^ splenic CD4^+^ or CD8^+^T cells pre-activated for 1 d by αCD3/CD28 Abs (columns 5–8 and 13–16) for another day. Keratinocytes remained unstimulated or were stimulated by IL-1α and/or TNF-α (30 ng/ml each), CsA (100 ng/ml), or IL-25 (2 μg/ml) as indicated. In the assays 7 and 15, 5 μg/ml of an Ab neutralizing IFN-γ was added. The supernatants of cultures were harvested and chemokine secretion was measured using a fluorescence-encoded bead-based assay (BioLegend), ^*^p < 0.05, ^**^p < 0.005, ^***^p < 0.001.

To determine the effect of lymphocytes on the secretion of chemokines by keratinocytes, we isolated primary keratinocytes from the tails of adult mice, and from newborn mice and cultured them in serum-free SFM keratinocyte medium (Gibco) supplemented with 0.06 mM Ca^++^. Under these conditions, which resemble those of the *stratum basale*, the keratinocytes rapidly formed a lawn of cobblestone-like cells. However, upon co-culture with splenic CD8^+^ or CD4^+^ T cells activated by αCD3/CD28 Abs for 1 d (or upon stimulation of keratinocytes with IL-1α, TNF-α, or IL-25) the tight layer of keratinocytes showed numerous holes in which T cells assembled (Supplementary Figures 1A–C). After 1 day of co-culture, no measurable differences were observed in cell viability and cell proliferation, as compared to individual cell cultures (Supplementary Figure 1D and data not shown). After one and 3 days of co-culture, the supernatant of cultures was collected to determine the secretion of 13 chemokines using a fluorescence-encoded bead-based assay (BioLegend). In addition, transcriptional changes of keratinocytes induced in the co-cultures were measured in NGS assays. Keratinocytes from adult or newborn mice cultured *in vitro* for 1 or 3 d secrete a restricted set of chemokines, mainly CXCL1 and CCL2, and very low amounts of CXCL5 (Figure 1, lanes 1, 9, and Supplementary Figure 2). Stimulation of adult keratinocytes with IL-1α and TNF-α enhanced CXCL1 and CCL2 secretion. Co-cultures of keratinocytes with pre-activated T cells resulted in an increase of CXCL5 secretion and also in the appearance of newly induced chemokines such as CCL3, CCL4, CXCL9 and CXCL10 (Figure 1, lanes 5-6 and 13-14**)**, which remained unaffected by adding CsA to the co-cultures (Figure 1, lanes 8/16).

The pattern of chemokines secreted by keratinocytes from the skin of newborn mice overlapped with that of adult tail keratinocytes but differed in detail. Remarkably, the stimuli that enhanced chemokine production diverged conspicuously between both types of keratinocytes. While IL-1α, TNF-α, and IL-25 affected mildly—or not at all—the chemokine production of adult tail keratinocytes, these stimuli strongly affected the secretion of CXCL5 and CCL20 by keratinocytes from newborn mice. Adding IL-1α and TNF-α to keratinocytes from newborn mice resulted in a 100 fold increase of CXCL5 and somewhat weaker of CCL20 secretion upon culture for 3 d (Supplementary Figure 2).

### CD8^+^T Cells Control Chemokine Secretion of Keratinocytes

In the co-cultures of T cells with keratinocytes, the same number (ratio 1:1) or 2-fold more CD8^+^T cells (i.e., ratio 2:1) pre-activated for 1 d by αCD3/CD28 Abs in X-vivo-medium were added. Since keratinocytes were maintained in serum-free keratinocyte medium this resulted in a 4:1 mix of keratinocyte: X-vivo media, respectively, and, thereby, in a drop of Ca^++^ in culture medium and subsequent silencing of numerous Ca^++^-dependent genes.

**Figure 2:**
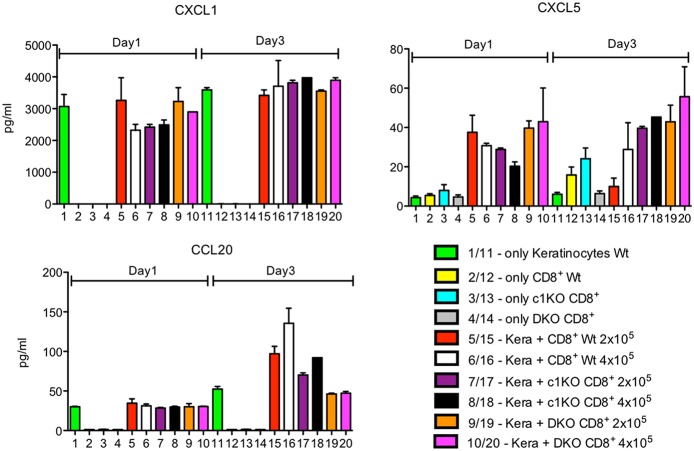
Secretion of chemokines CXCL1, CXCL5 and CCL20 by keratinocytes alone or upon co-culture with CD8^+^T cells. Keratinocytes cultured for 7 d *in vitro* and splenic CD8^+^ T cells pre-activated by αCD3/CD28 Ab for 1 d were either cultured alone or together in 6-well plates for 1 or 3 d as indicated and their chemokine secretion measured.

The secretion of CXCL1 and CCL2 chemokines remained either unaffected or was slightly decreased while that of CXCL5 and CCL20 was increased upon co-culture with CD8^+^T cells (Figures 1, 2). This is especially the case in wild type and NFATc1-ko CD8^+^T cells. T cells from mice double ko (DKO) for NFATc1 and NFATc2 remained “neutral” with respect to CXCL1 and CCL20 secretion and enhanced slightly the expression of CXCL5 when compared to wild type and NFATc1-ko CD8^+^T cells (Figure 2). Since DKO CD8^+^T cells are prone to apoptosis one may speculate that pro-apoptotic signals emerged from those cells to increase the secretion of pro-inflammatory chemokine CXCL5 (19) by keratinocytes.

**Figure 3:**
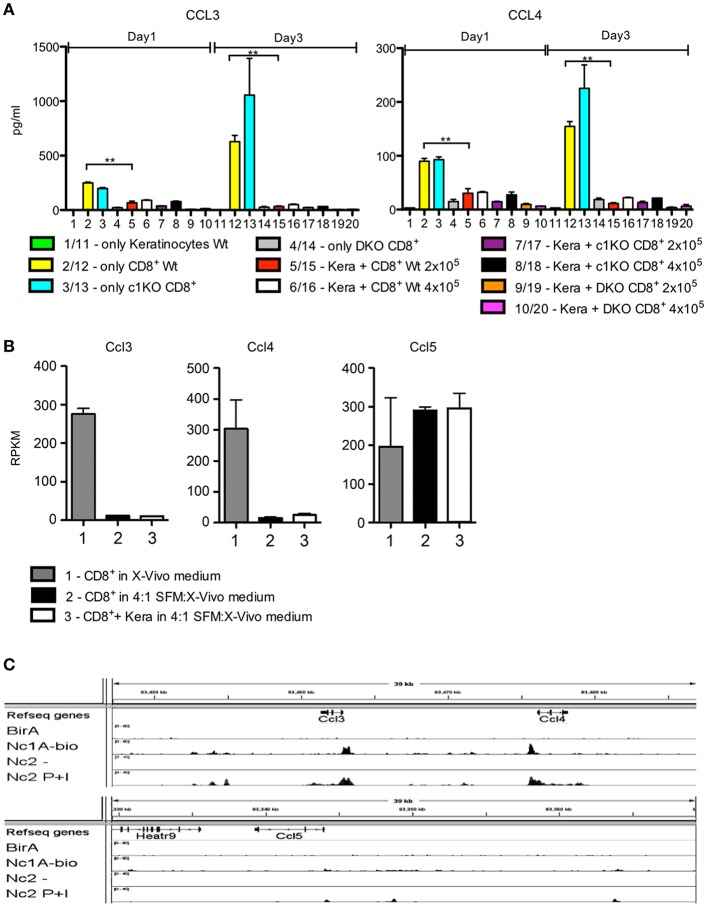
Silencing of CCL3 and CCL4 secretion in keratinocyte medium. **(A)** Secretion of chemokines CCL3 and CCL4 by keratinocytes, CD8^+^T cells, or co-cultures of both. Keratinocytes cultured for 7 d *in vitro* and splenic CD8^+^ T cells pre-activated by αCD3/CD28 Ab for 1 d were either cultured alone or together in 6-well plates for 1 or 3 d as indicated and their chemokine secretion was measured. **(B)** Suppression of *Ccl3* and *Ccl4*, but not of *Ccl5* transcription upon culture of pre-activated CD8^+^T cells for 1 d in keratinocyte/X-vivo medium (4:1) alone (column 2) or in the presence of keratinocytes (column 3). Column 1 shows the RNA levels of genes in pre-activated CD8^+^T cells cultured in X-vivo medium for 1 d. **(C)** Binding of NFATc1-bio protein to the promoters of murine *Ccl3* and *Ccl4* genes in activated cytotoxic T cells. Results of ChIP seq assays (16). Splenic CD8^+^T cells from transgenic mice expressing NFATc1-bio proteins were stimulated by T+I for 5 h. Their chromatin was cross-linked by 4% formaldehyde-treatment of cells *in vitro*, followed by shearing of chromatin and binding to streptavidin beads. After the release of DNA from cross-linked chromatin complexes, DNA was sequenced in NGS assays (16, 20), ^*^p < 0.05, ^**^p < 0.005, ^***^p < 0.001.

The secretion of CCL3 and CCL4 by CD8^+^T cells was strongly suppressed upon co-culture with keratinocytes or upon culture in SFM medium (Figure 3A). This silencing effect was also reflected at the transcriptional level. The strong transcription of *Ccl3* and *Ccl4* genes in pre-activated CD8^+^T cells was almost abolished upon culture of T cells in SFM/X-vivo (4:1) medium, without or with co-culture with keratinocytes whereas that of the *Ccl5* gene remained unaffected (Figure 3B). This is due to the defect of NFAT activity in low Ca^++^ medium. In activated cytotoxic T cells the promoters of *Ccl3* and *Ccl4* genes – but not of the *Ccl5* gene—are bound by NFATc1 (Figure 3C) and, therefore, are excellent targets for NFATc1 in cytotoxic T cells (16).

### CXCL9 and CXCL10 Are Strongly Induced in Keratinocytes by IFN-γ at the Transcriptional Level

**Figure 4:**
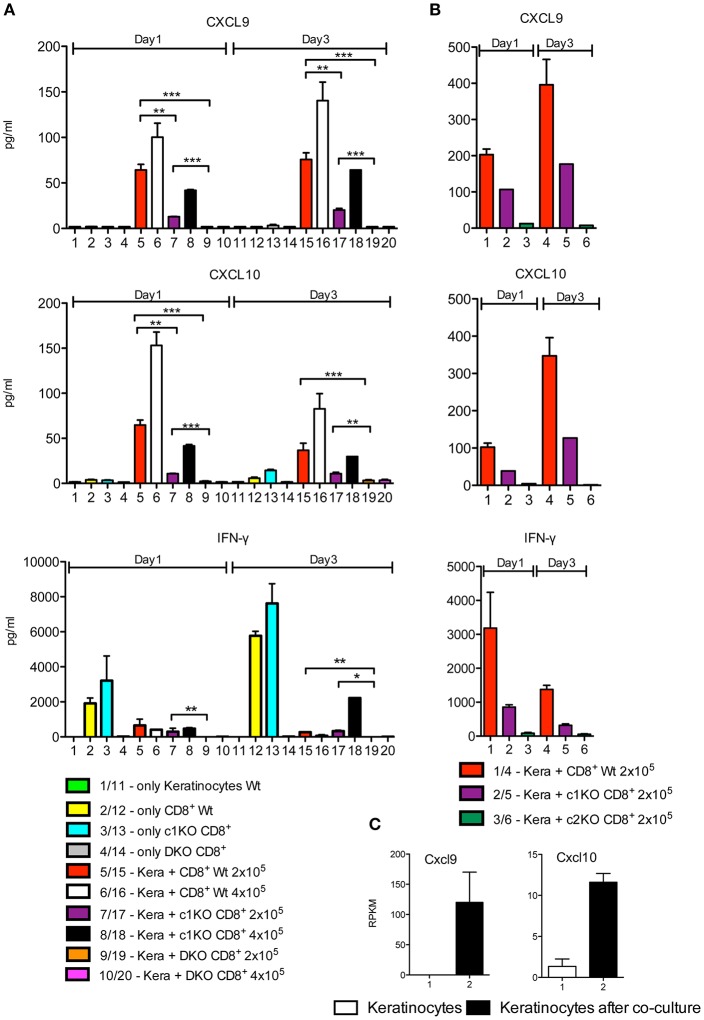
Induction of CXCL9, CXCL10 and IFN-γ secretion in keratinocytes upon co-culture with CD8^+^T cells. **(A,B)** Secretion of chemokines CXCL9 and CXCL10 and of IFN-γ by keratinocytes and CD8^+^T cells. Keratinocytes cultured for 7 d *in vitro* and splenic CD8^+^ T cells pre-activated by αCD3/CD28 Ab for 1 d were either cultured alone or together in 6-well plates for another 1 or 3 d as indicated and their chemokine and IFN-γ secretion measured. In **(A)** the incubation of NFAT-DKO T cells was included, in **(B)** the incubation of NFATc2-ko instead of DKO T cells. **(C)** Induction of *Cxcl9* and *Cxcl10* transcription in keratinocytes that were cultured alone (column 1) or together with the same number of pre-activated CD8^+^ T cells for 1 d (column 2). In the co-culture, the CD8^+^ T cells were washed off, and the RNA levels of keratinocytes were determined in NGS assays, ^*^p < 0.05, ^**^p < 0.005, ^***^p < 0.001.

CXCL9 and CXCL10 were neither secreted by untreated keratinocytes nor by activated WT CD8^+^T cells. However, upon co-culture of keratinocytes with T cells pre-activated by αCD3/CD28 for 1 d a massive induction of both chemokines was observed. This was correlated with the NFAT expression of T cells, since NFAT DKO CD8^+^T cells were unable to induce any CXCL9 and CXCL10 secretion and NFATc2–ko CD8^+^T cells showed a marked drop in the induction of both chemokines. In contrast, in three out of five co-culture assays NFATc1-ko cells did not show any restriction in chemokine induction whereas in two assays a decrease was detected (Figures 4A,B and Supplementary Figure 3A). A strong induction of CXCL9 and CXCL10 chemokines was also observed in keratinocytes after culture for 1 d with “T cell-conditioned medium,” i.e., with X-VIVO medium in which CD8^+^T cells were activated for 1 d by αCD3/CD28, whereas a poor induction was observed using conditioned medium from NFAT DKO CD8^+^T cells (Supplementary Figure 3B). The differences in chemokine induction could be explained by marked differences in IFN-γ secretion between NFAT DKO, NFATc1-ko, and NFATc2-ko CD8^+^T cells. While DKO CD8^+^T cells did not synthesize any IFN-γ, NFATc2-ko showed a strong, and NFATc1-ko cells a more moderate decrease in IFN-γ secretion before and after co-culture (Figures 4A,B). Along with the suppressive effect of Abs directed against IFN-γ on CXCL9 and CXCL10 secretion (Figure 1 and Supplementary Figure 2), these data indicate that IFN-γ produced by CD8^+^T cells stimulates keratinocytes to secrete CXCL9 and CXCL10.

NGS assays of the transcriptome of keratinocytes from WT mice before and after co-culture with T cells for 1 d demonstrated that the induction of CXCL9 and CXCL10 secretion occurs at the transcriptional level. In cultured murine keratinocytes, 8440 genes were expressed in 5 and more RPKM, and 503 genes were changed 2.5 fold and more in their expression level between keratinocytes without or upon co-culture with T cells. When these 503 genes were analyzed using the GORILLA (gene ontology enrichment analysis and visualization) tool, among the most prominent 10 gene sets, 9 corresponded to genes controlling response and defense mechanisms (Table 1). While the top 2 pathways comprise genes induced in the interferon-beta response, including the genes encoding the transcription factors IRF1 and STAT1, the other pathways are characterized by further response genes, including the *Cxcl9* and *Cxcl10* genes (Supplementary Table 1). Upon co-culture for 1 d, the RNA levels of the *Cxcl9* gene increased more than 100 fold, and that of the *Cxcl10* gene approximately 10 fold (Figure 4C). A 2–5 fold increase was detected for the two genes encoding STAT1 and STAT2. A similar increase was observed for the *Cd274* gene encoding PD1 ligand and the *Tnf* and *Cd40lg* genes (Supplementary Figure 3C) that defend keratinocytes against T cell-mediated killing.

It is worth mentioning that within the gene sets of the top 10 pathways induced in keratinocytes by T cells the overwhelming majority of genes corresponded to genes induced by type I interferons. In addition to the genes encoding the transcription factors STAT1, STAT2, IRF1, IRF7 and IRF8, these include the *Casp1* and *Gsdmd* genes that code for caspase-1 and gasdermin D—prominent inducers of pyroptosis and membrane pore formation (21)—and numerous genes coding for guanylate binding proteins, Tap transporters and proteasome subunits. All these genes are compiled in the Supplementary Table 1.

### Local CXCL10 Induction in Keratinocytes From Lichen Planus Patients

**Figure 5:**
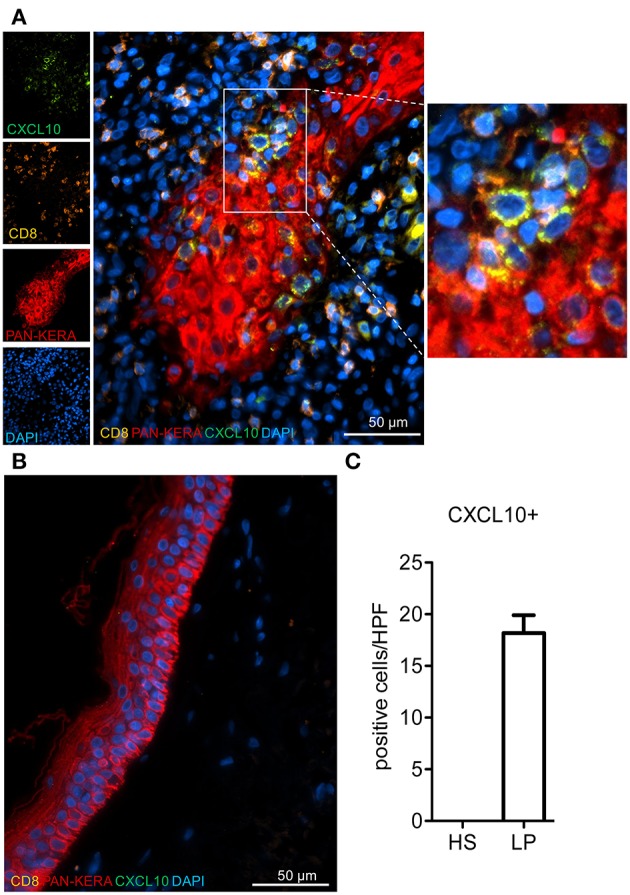
Enumeration of selectively accentuated CXCL10+ keratinocytes in lichen planus patients. Representative immunofluorescence stainings for CXCL10, CD8, and pan-cytokeratin (Pan-Kera) in **(A)** lichen planus (LP) and **(B)** healthy skin (HS). Rectangle marks position of blow-up (right side). **(C)** CXCL10^+^ cells were counted in at least 3 high power fields (HPF)/patient (*n* = 10) and compared to HS. Confocal microscopy, 40x magnification.

Finally, we wanted to see whether our *in vitro/ex vivo* findings on the interplay between murine CD8^+^T cells and keratinocytes are of relevance for human skin disorders. To this end, we selected lichen planus (LP) as model disease. LP is a T cell mediated inflammatory disease that is characterized by the massive infiltration of T lymphocytes, the destruction of the basal epidermal layer and the induction of numerous genes with a typical IFN signature (12, 13, 22). These include the chemokine *Cxcl9* and *Cxcl10* genes as typical molecular LP markers that distinguish lichenoid tissue reactions from atopic dermatitis and psoriasis (12). In our co-stains of LP skin sections using Abs directed against CXCL10, CD8, CD4, and pan-keratin (or Krt14) we detected a strong expression of CXCL10 in keratinocytes located mostly at the edge of elongated epidermal ridges closely surrounded by infiltrating T cells. In contrast, infiltrating CD8^+^ and CD4^+^T cells showed rather a very weak staining with the CXCL10 Ab (Figure 5 and Supplementary Figures 4, 5). For quantification of CXCL10-expressing keratinocytes we counted positive cells in each immunofluorescence stain of 10 patients and compared results with healthy skin (Figure 5).

**Figure 6:**
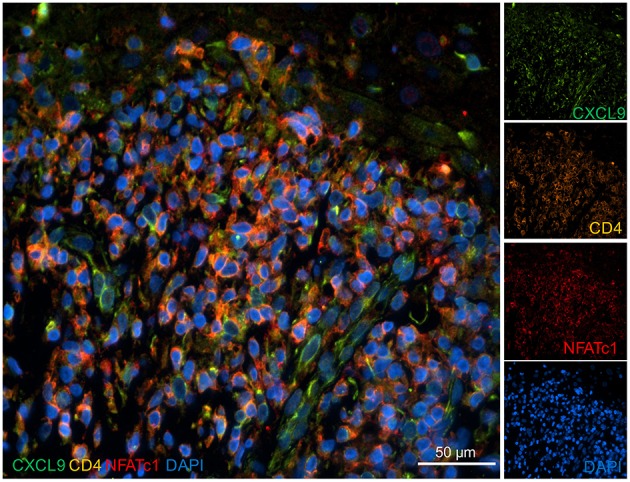
Expression of CXCL9 in the skin of a lichen planus patient. Skin sections were stained with Abs directed against CXCL9, NFATc1, and CD4 and co-stained with DAPI as indicated. Immunofluorescence microscopy, 40x magnification.

The immunofluorescence stains with two Abs directed against CXCL9 did not show a distinct expression of CXCL9 in keratinocyte at the keratinocyte/T cell border but a broad, more diffuse expression in the invading cells, including CD4^+^T lymphocytes and other cells (Figure 6).

The data on the role of NFAT factors in CXCL9 and CXCL10 induction prompted us to speculate that in lichenoid tissue reactions the nuclear expression of NFATs might be a further molecular sign of infiltrating T cells. However, in all immunofluorescence stains of such skin sections we were unable to detect any clear-cut nuclear NFATc1 or NFATc2 localization in infiltrating CD8^+^ and CD4^+^ T cells, but a prominent cytosolic expression of both NFAT factors (Supplementary Figures 6A,B).

## Discussion

The experimental data presented here extend a large body of published results on the interplay between epidermal keratinocytes and T lymphocytes (23, 24). Employing co-culture experiments and analyzing supernatants (“conditioned medium”) of activated CD8^+^T cells we demonstrated that T lymphocytes modulate the chemokine synthesis of keratinocytes, including the IFN-γ-mediated induction of CXCL9 and CXCL10 chemokines. In addition, we detected the induction of a large set of type I IFN-inducible genes in keratinocytes. Similar changes in gene expression have been described in the skin of lichen planus patients (13) in which we detected the distinct expression of CXCL10 in keratinocytes near the border to invading T cells.

Compared to the situation *in vivo*, there are limitations of our co-culture system. The infiltration of lymphocytes into the skin and the attack of the stratum basale are a feature of many skin diseases. In our co-cultures, keratinocytes of stratum basale were exclusively propagated and studied. Therefore, the attack of pre-activated T cells and the “liquefaction degeneration” of the basal layer of the epidermis by cytotoxic lymphocytes might reflect certain pathological situations *in vivo*, such as in the skin of patients suffering from lichen planus.

The massive changes in keratinocyte gene expression induced by T cells suggest the existence of a fine-tuned balance between keratinocytes and T lymphocytes that is disturbed in numerous skin diseases. This might be due to metabolic changes in primary keratinocytes which, like activated CD8^+^T cells (16), exhibit a high glycolysis rate (own unpublished data). Our data indicate that CXCL9 and CXCL10 are induced by IFN-γ secreted by T cells. Both chemokines attract (autoreactive) T cells expressing CXCR3, the common receptor for CXCL9, CXCL10 and CXCL11 (4), to the skin (25, 26). Albeit the co-culture system does not reflect the complex cellular interplay between keratinocytes, T cells, macrophages, dendritic cells, and others in human skin diseases, it contributes to the understanding of T cells/keratinocytes interactions. In LP patients, this “feed-back” mechanism leads to a “flood” of invading T lymphocytes into the dermis, the liquefaction degeneration of the basal epidermal layer and the interference with keratinocyte differentiation (27). Surprisingly, unlike CXCL10 we were unable to detect a distinct expression of CXCL9 in keratinocytes of LP patients, in spite of the comparable induction of CXCL9 and CXCL10 secretion by murine keratinocytes upon co-culture with CD8^+^T cells. However, a similar dichotomy in the expression of CXCL9 and CXCL10 (and CXCL11) has been described before for different human skin disorders. In those studies CXCL10 and CXCL11 were found to be predominantly expressed in basal keratinocytes whereas CXCL9 expression was predominantly localized in dermal infiltrates (15, 28).

It should be noted here that CXCR3 is not a skin-specific chemokine receptor but attracts immune cells to sites of IFN-γ-mediated inflammation (3). CXCR3 is not only expressed on activated T cells but also on dendritic cells, natural killer cells, fibroblasts, smooth muscle, and epithelial cells (25, 29). Accordingly, the expression of CXCR3 and its activation by chemokine ligands has been associated not only with numerous skin diseases but also with other disorders, such as arthritis, type 1 diabetes (30, 31), and allograft rejections including the rejection of heterotopic heart allografts (32, 33). These findings underline the importance of CXCL9/CXCL10 signals in the control of (auto-) immunity and have been exploited in immune therapies against tumors (3, 34) in which CXCR3 is strongly expressed (35, 36).

Apart from CXCL9 and CXCL10, the induction of genes encoding caspase-1 and gasdermin D are additional steps in the T cell-mediated “killing program” of keratinocytes. Caspase-1 is an inflammatory response initiator that cleaves other proteins, including the pyroptosis inducer gasdermin D (21). However, keratinocytes are also able to fight back, as it is shown by the induction of the *Cd274* gene coding for the PD1 ligand, which—through its binding to PD-1 on activated T cells—dampens T cell activity (37, 38). Most of these genes are induced via type I IFN pathways. This is demonstrated by the induction of *Irf* genes *Irf1, Irf7*, and *Irf8*, and of Stat factors STAT1 and STAT2 that are induced by type I IFNs (Supplementary Table 1).

The decrease in expression of *Ccl3* and *Ccl4* genes in pre-activated CD8^+^T cells that we observed upon culture of T cells in Ca^++^-poor SFM/X-vivo medium might be of relevance for the fate of T cells infiltrating the skin. In the basal layer of the epidermis extracellular Ca^++^ concentrations are very low and facilitate the proliferation of keratinocytes whereas increasing Ca^++^ supports keratinocyte differentiation (39, 40). Therefore, one may speculate that under physiological conditions invading T cells that need Ca^++^ for full activity are inhibited. This is reflected in the suppression of numerous Ca^++^-dependent genes in our assays, including *Ccl3, Ccl4, Ifn-*γ, and further NFAT-dependent genes.

The need for T cell activation and the inability of NFAT DKO T cells to induce CXCL9 and CXCL10 indicate that the IFN-γ-mediated induction of both chemokines is linked to NFAT activity in T cells. In activated CD8^+^ T cells and, *in vivo*, in cytotoxic T cells the synthesis of IFN-γ is under the control of the NFAT factors NFATc1 and NFATc2. In CTLs, inactivation of NFATc1 led to a complete loss of IFN-γ induction upon TPA+ionomycin treatment whereas NFATc2 inactivation exerted a milder defect, leading to a reduction of ~60% (16). Therefore, we expected to see a strong defect in CXCL9 and CXCL10 induction by NFATc1-ko, but not NFATc2-ko T cells. However, the opposite was observed. The strong decrease in IFN-γ production by NFATc2-ko CD8^+^T cells suggests that under the chosen experimental conditions, i.e., 1 day activation of splenic CD8^+^T cells by αCD3/CD28 (followed by co-culture with keratinocytes for 1 d), NFATc2—and not NFATc1—is the most prominent NFAT factor to stimulate IFN-γ expression in murine CD8^+^T cells. It remains to be shown whether this is also true for skin-infiltating T cells in human skin diseases.

## Data Availability

The datasets generated for this study can be found in the data of NGS assays have been deposited in the GEO database (GSE131905).

## Ethics Statement

Animal experiments were performed according to project licenses (Nr. 55.2-2531.01-80/10), which are approved and controlled by the Regierung von Unterfranken, Würzburg. All anonymized specimens from LP patients were obtained from the archive of paraffin-embedded diagnostic tissues of the Department of Dermatology at the University Hospital of Würzburg, Germany, and were used in accordance with the local ethics committee.

## Author Contributions

TR, VS, MA, SK-H, and KMuh: Performed experiments. KMur, FG, and AK: Immunofluorescence stains. KMuh, MK, and TB: NGS assays. MG and AK: Analyzed data and supported the preparation of the manuscript. KMuh and ES: Led the investigation and wrote the manuscript.

### Conflict of Interest Statement

The authors declare that the research was conducted in the absence of any commercial or financial relationships that could be construed as a potential conflict of interest.

## Abbreviations

CsA: cyclosporine A
CTL: cytotoxic T cells
DKO T cells: T cells deficient for NFATc1 and NFATc2
Interferon-γ: IFN-γ
LP: lichen planus
NGS: next generation sequencing
TNF-α: Tumor necrosis factor α
T+I: TPA and ionomycin
IFN: Type I interferon.
