# Supplementary material for: T Cells Control Chemokine Secretion by Keratinocytes
Source: Front Immunol. 2019 Aug 9;10:1917. doi: 10.3389/fimmu.2019.01917 (PMC6696622; doi:10.3389/fimmu.2019.01917)
Supplement: Supplementary file 1 [file Table_1.pdf]

Supplementary Table 1: List of individual genes induced in murine keratinocytes upon co-culture with pre-activated CD8$^+$ T cells for 1 d, determined with the Gorilla (**G**ene **O**ntology en**RI**chment ana**L**ysis and visualization) tool [1]. The chemokines Cxcl9 and Cxcl10 are underlined and written in bold face letters.

**<u>GO:0035456</u>: response to interferon-beta**<br>*Tgtp2* - t cell specific gtpase 2; *Gbp6* - guanylate binding protein 6; *Irf1* - interferon regulatory factor 1; *Gm4841* - predicted gene 4841;*Bst2* - bone marrow stromal cell antigen 2; *Gbp3* - guanylate binding protein 3; *Irgm2* - immunity-related gtpase family m member 2;*Mnda* - myeloid cell nuclear differentiation antigen*;* *Ifit3* - interferon-induced protein with tetratricopeptide repeats 3;*Gbp2* - guanylate binding protein 2*;* *Ifi205* - interferon activated gene 205*;* *Ifi47* - interferon gamma inducible protein 47;*Ifit1* - interferon-induced protein with tetratricopeptide repeats 1; *Igtp* - interferon gamma induced gtpase; *Ifitm3* - interferon induced transmembrane protein 3; *Ifi204* - interferon activated gene 204*;* *Irgm1* - immunity-related gtpase family m member 1; *Iigp1* - interferon inducible gtpase 1; *Tgtp1* - t cell specific gtpase 1;*Stat1* - signal transducer and activator of transcription 1
**<u>GO:0035458</u>: cellular response to interferon-beta**<br>*Tgtp2* - t cell specific gtpase 2; *Gbp6* - guanylate binding protein 6; *Gm4841* - predicted gene 4841;; *Irf1* - interferon regulatory factor 1; *Gbp3* - guanylate binding protein 3; *Mnda* - myeloid cell nuclear differentiation antigen; *Irgm2* - immunity-related gtpase family m member 2; *Gbp2* - guanylate binding protein 2; *Ifit3* - interferon-induced protein with tetratricopeptide repeats 3; *Ifi205* - interferon activated gene 205; *Ifi47* - interferon gamma inducible protein 47; *Ifit1* - interferon-induced protein with tetratricopeptide repeats 1; *Igtp* - interferon gamma induced gtpase; *Ifi204* - interferon activated gene 204; *Irgm1* -immunity-related gtpase family m member 1; *Iigp*1 - interferon inducible gtpase 1; *Tgtp1* - t cell specific gtpase 1; *Stat1* - signal transducer and activator of transcription 1
**<u>GO:0051707</u>: response to other organism**<br>*Gbp7* - guanylate binding protein 7; *Gbp6* - guanylate binding protein 6; *Samhd1* - sam domain and hd domain, 1; *Irf8* - interferon regulatory factor 8;*Bst2* - bone marrow stromal cell antigen 2; *Gbp3* - guanylate binding protein 3; <u>***Cxcl9*** **- chemokine (c-x-c motif) ligand 9**</u>; *Irgm2* - immunity-related; gtpase family m member 2; *Mnda* - myeloid cell nuclear differentiation antigen; *Oas3* - 2'-5' oligoadenylate synthetase 3; *Ly6a* - lymphocyte antigen 6 complex, locus a; *Ifit1* - interferon-induced protein with tetratricopeptide repeats 1; *Isg15* - isg15 ubiquitin-like modifier; *Ifi204* - interferon activated gene 204; *Irgm1* - immunity-related gtpase family m member 1; *Rtp4* - receptor transporter protein 4; <u>***Cxcl10*** **- chemokine (c-x-c motif) ligand 10**</u>; *Iigp1* - interferon inducible gtpase 1; *Irf7* - interferon regulatory factor 7; *Serpinb9* - serine (or cysteine) peptidase inhibitor, clade b, member 9; *Dtx3l* - deltex 3-like (drosophila); *Plac8* - placenta-specific 8; *Zbp1* - z-dna binding protein 1; *Lgals9* - lectin, galactose binding, soluble 9; *Irf1* - interferon regulatory factor 1; *Casp1* - caspase 1; *Trim12a* - tripartite motif-containing 12a; *Ifit3* - interferon-induced protein with; tetratricopeptide repeats 3; *Gbp2* - guanylate binding protein 2; *Ifi205* - interferon activated

gene 205;*AA467197*-expressed sequence aa467197;*Parp9* - poly (adp-ribose) polymerase family, member 9; *Ifitm3* - interferon induced transmembrane protein 3; *Oasl2* - 2'-5' oligoadenylate synthetase-like 2; *Tnf* - tumor necrosis factor; *Lsm5* - lsm5 homolog, u6 small nuclear rna associated (s. cerevisiae); *Nlrc5* - nlr family, card domain containing 5; *Stat2* - signal transducer and activator of transcription 2; *Tgtp1* - t cell specific gtpase 1; *Psmb9* - proteasome (prosome, macropain) subunit, beta type 9 (large multifunctional peptidase 2); *Stat1* - signal transducer and activator of transcription 1; *Ppbp* - pro-platelet basic protein
**GO:0043207: response to external biotic stimulus**<br>*Gbp7* - guanylate binding protein 7; *Gbp6* - guanylate binding protein 6; *Samhd1* - sam domain and hd domain, 1; *Irf8* - interferon regulatory factor 8; *Bst2* - bone marrow stromal cell antigen 2; *Gbp3* - guanylate binding protein 3; Irgm2 - immunity-related gtpase family m member 2; ***Cxcl9* - chemokine (c-x-c motif) ligand 9**; *Cd274* - cd274 antigen; *Mnda* - myeloid cell nuclear differentiation antigen; *Oas3* - 2'-5' oligoadenylate synthetase 3; *Ly6a* - lymphocyte antigen 6 complex, locus a; *Ifit1* - interferon-induced protein with tetratricopeptide repeats 1; *Isg15* - isg15 ubiquitin-like modifier; *Ifi204* - interferon activated gene 204; *Irgm1* - immunity-related gtpase family member 1; *Rtp4* - receptor transporter protein 4; ***Cxcl10* - chemokine (c-x-c motif) ligand 10**; *Iigp1* - interferon inducible gtpase 1; *Irf7* - interferon regulatory factor 7; *Serpinb9* - serine (or cysteine) peptidase inhibitor, clade b, member 9; *Dtx3l* - deltex 3-like (drosophila); *Plac8* - placenta-specific 8; *Pde2a* - phosphodiesterase 2a, cgmp-stimulated; *Zbp1* - z-dna binding protein 1; *Lgals9* - lectin, galactose binding, soluble 9; *Irf1* - interferon regulatory factor 1; *Casp*1 - caspase 1; *Trim12a* - tripartite motif-containing 12a; *Ifit3* - interferon-induced protein with tetratricopeptide repeats 3; *Gbp2* - guanylate binding protein 2; *Tap2* - transporter 2, atp-binding cassette, sub-family b (mdr/tap); *Ifi205* - interferon activated gene 205; *AA467197* - expressed sequence aa467197; *Parp9* - poly (adp-ribose) polymerase family, member 9; *Ifitm3* - interferon induced transmembrane protein 3; *Oasl2* - 2'-5' oligoadenylate synthetase-like 2; *Tnf* - tumor necrosis factor; *Lsm5* - lsm5 homolog, u6 small nuclear rna associated (s. cerevisiae); *Nlrc5* - nlr family, card domain containing 5; *Stat2* - signal transducer and activator of transcription 2; *Tgtp1* - t cell specific gtpase 1; *Psmb9* - proteasome (prosome, macropain) subunit, beta type 9 (large multifunctional peptidase 2); *Stat1* - signal transducer and activator of transcription 1;*Ppbp* - pro-platelet basic protein
**GO:0006952: defense response**<br>*Gbp7* - guanylate binding protein 7; *Gbp6* - guanylate binding protein 6; *Samhd1* - sam domain and hd domain, 1; *Irf8* - interferon regulatory factor 8; *Bst2* - bone marrow stromal cell antigen 2; *Gbp3* - guanylate binding protein 3; *Trim21* - tripartite motif-containing 21; *Irgm2* - immunity-related gtpase family m member 2; ***Cxcl9* - chemokine (c-x-c motif) ligand 9**; *Ifi47* - interferon gamma inducible protein 47; *Ifit1* - interferon-induced protein with tetratricopeptide repeats 1; *Igtp* - interferon gamma induced gtpase;*Isg15* - isg15 ubiquitin-like modifier; *Irgm1* - immunity-related gtpase family m member 1; *Rtp4* - receptor transporter protein 4; ***Cxcl10* - chemokine (c-x-c motif) ligand 10**;*Iigp1* - interferon inducible gtpase 1; *Irf7* - interferon regulatory factor 7; *Parp14* - poly (adp-ribose) polymerase family, member 14; *Dtx3l* - deltex 3-like (drosophila); *Tgtp2* - t cell specific gtpase 2; *Zbp1* - z-dna binding protein 1; *Gm4841* - predicted gene 4841; *Irf1* - interferon regulatory factor 1; *Serping1* - serine (or cysteine) peptidase inhibitor, clade g, member 1; *Gbp2* - guanylate binding protein 2; *Ifit3* - interferon-induced protein with tetratricopeptide repeats;3 *C1ra* - complement component 1, r subcomponent a; *Tap1* - transporter 1, atp-binding cassette, sub-family b (mdr/tap); *Parp9* - poly (adp-ribose) polymerase family,

member 9; *Ifitm3* - interferon induced transmembrane protein 3;*Oasl2* - 2'-5' oligoadenylate synthetase-like 2; *Tnf* - tumor necrosis factor;*Tgtp1* - t cell specific gtpase 1; *Nlrc5* - nlr family, card domain containing 5; Stat1 - signal transducer and activator of transcription 1; Ppbp - pro-platelet basic protein
**<u>GO:0009607</u>: response to biotic stimulus**<br>*Gbp7* - guanylate binding protein 7; *Gbp6* - guanylate binding protein 6; *Samhd1* - sam domain and hd domain, 1; *Irf8* - interferon regulatory factor 8; *Bst2* - bone marrow stromal cell antigen 2; *Gbp3* - guanylate binding protein 3; ***<u>Cxcl9</u>* <u>- chemokine (c-x-c motif) ligand 9</u>**; *Irgm2* - immunity-related gtpase family m member 2; *Cd274* - cd274 antigen; *Mnda* - myeloid cell nuclear differentiation antigen; *Oas3* - 2'-5' oligoadenylate synthetase 3; *Ly6a* - lymphocyte antigen 6 complex, locus a; *Ifit1* - interferon-induced protein with tetratricopeptide repeats 1; *Isg15* - isg15 ubiquitin-like modifier; *Ifi204* - interferon activated gene 204; *Irgm1* - immunity-related gtpase family m member 1; *Rtp4* - receptor transporter protein 4; ***<u>Cxcl10</u>* <u>- chemokine (c-x-c motif) ligand 10</u>**; *Iigp1* - interferon inducible gtpase 1; *Irf7* - interferon regulatory factor 7; *Serpinb9* - serine (or cysteine) peptidase inhibitor, clade b, member 9; *Dtx3l* - deltex 3-like (drosophila); *Plac8* - placenta-specific 8; *Pde2a* - phosphodiesterase 2a, cgmp-stimulated; *Zbp1* - z-dna binding protein 1; *Lgals9* - lectin, galactose binding, soluble 9; *Casp1* - caspase 1;*Irf1* - interferon regulatory factor 1; *Trim12a* - tripartite motif-containing 12a; *Ifit3* - interferon-induced protein with tetratricopeptide repeats 3; *Gbp2* - guanylate binding protein 2; *Tap2* - transporter 2, atp-binding cassette, sub-family b (mdr/tap); *Ifi205* - interferon activated gene 205; *AA467197* - expressed sequence aa467197; *Parp9* - poly (adp-ribose) polymerase family, member 9; *Ifitm3* - interferon induced transmembrane protein 3; *Oasl2* - 2'-5' oligoadenylate synthetase-like 2; *Tnf* - tumor necrosis factor; *Lsm5* - lsm5 homolog, u6 small nuclear rna associated (s. cerevisiae); *Nlrc5* - nlr family, card domain containing 5; *Stat2* - signal transducer and activator of transcription 2; *Tgtp1* - t cell specific gtpase 1; *Psmb9* - proteasome (prosome, macropain) subunit, beta type 9 (large multifunctional peptidase 2); *Stat1* - signal transducer and activator of transcription 1; *Ppbp* - pro-platelet basic protein
**<u>GO:0009617</u>: response to bacterium**<br>*Gbp7* - guanylate binding protein 7; *H2-T23* - histocompatibility 2, t region locus 23; *Sp110* - sp110 nuclear body protein; *Gbp6* - guanylate binding protein 6; *Mnda* - myeloid cell nuclear differentiation antigen; *Irgm2* - immunity-related gtpase family m member 2; ***<u>Cxcl9</u>* <u>- chemokine (c-x-c motif) ligand 9</u>**; *Ly6a* - lymphocyte antigen 6 complex, locus a; *Fcer1g* - fc receptor, ige, high affinity i, gamma polypeptide; *Hmgb2* - high mobility group box 2; *H2-M3* - histocompatibility 2, m region locus 3; *Gpx1* - glutathione peroxidase 1; *Gpx2* - glutathione peroxidase 2; *Ppp1r11* - protein phosphatase 1, regulatory (inhibitor) subunit 11; *Serpinb9* - serine (or cysteine) peptidase inhibitor, clade b, member 9; *Pycard* - pyd and card domain containing;*Tmem229b* - transmembrane protein 229b; *Dhx58* - dexh (asp-glu-x-his) box polypeptide 58; *Gbp2* - guanylate binding protein 2; *AA467197* - expressed sequence aa467197; *Ifi205* - interferon activated gene 205; *Ifi44* - interferon-induced protein 44; *Hba-a2* - hemoglobin alpha, adult chain 2; *Tnf* - tumor necrosis factor; *Stat1* - signal transducer and activator of transcription 1; *Psmb9* - proteasome (prosome, macropain) subunit, beta type 9 (large multifunctional peptidase 2); *Irf8* - interferon regulatory factor 8; *Gbp3* - guanylate binding protein 3; *Ifit1* - interferon-induced protein with tetratricopeptide repeats 1; *Isg15* - isg15 ubiquitin-like modifier; *Ifi204* - interferon activated gene 204; *Irgm1* - immunity-related gtpase family m member 1; ***<u>Cxcl10</u>* <u>- chemokine (c-x-c motif) ligand 10</u>**; *Tslp* - thymic stromal lymphopoietin; *Iigp1* - interferon inducible gtpase 1; *Coch* - coagulation factor c homolog (limulus polyphemus); *Serpina3f* - serine (or cysteine) peptidase inhibitor, clade a, member 3f; *Plac8* - placenta-specific 8;

Slfn2 - schlafen 2; *Ifng* - interferon gamma; *Casp1* - caspase 1; *Myd88* - myeloid differentiation primary response gene 88; *Ifit3* - interferon-induced protein with tetratricopeptide repeats 3; *Tnfaip8* - tumor necrosis factor, alpha-induced protein8;*H2-K1*- histocompatibility2,k1,k region *Defb3* - defensin beta 3;*Mndal* - myeloid nuclear differentiation antigen like; *Gsdmd* - gasdermin d; *Lsm5* - lsm5 homolog, u6 small nuclear rna associated (s. cerevisiae); *Nlrc5* - nlr family, card domain containing 5; *Tgtp1* - t cell specific gtpase 1
**GO:0002376: immune system process**<br>*Samhd1* - sam domain and hd domain, 1; *Nup85* - nucleoporin 85; *Mnda* - myeloid cell nuclear differentiation antigen; ***Cxcl9* - chemokine (c-x-c motif) ligand 9**; *Irgm2* - immunity-related gtpase family m member 2; *Oas3* - 2'-5' oligoadenylate synthetase 3; *H2-T22* - histocompatibility 2, t region locus 22; *Ahcy* - s-adenosylhomocysteine hydrolase; *Nampt* - nicotinamide phosphoribosyltransferase; *Rtp4* - receptor transporter protein 4; *Tinagl1* - tubulointerstitial nephritis antigen-like 1; *Serpinb9* - serine (or cysteine) peptidase inhibitor, clade b, member 9; *Parp14* - poly (adp-ribose) polymerase family, member 14; *Erap1* - endoplasmic reticulum aminopeptidase 1; *Il18bp* - interleukin 18 binding protein; *Psme2* - proteasome (prosome, macropain) activator subunit 2 (pa28 beta); *Trim12a* - tripartite motif-containing 12a; *Psme1* - proteasome (prosome, macropain) activator subunit 1 (pa28 alpha); *Tap2* - transporter 2, atp-binding cassette, sub-family b (mdr/tap); *Ifi205*-interferon activated gene 205 *Tap1* - transporter 1, atp-binding cassette, sub-family b (mdr/tap); *Oasl2* - 2'-5' oligoadenylate synthetase-like 2; *Tapbp* - tap binding protein;*Tnf* - tumor necrosis factor; *Stat2* - signal transducer and activator of transcription 2; *Stat1* - signal transducer and activator of transcription 1;*Psmb9* - proteasome (prosome, macropain) subunit, beta type 9 (large multifunctional peptidase 2); *Psmb8* - proteasome (prosome, macropain) subunit, beta type 8 (large multifunctional peptidase 7); *Psmb10* - proteasome (prosome, macropain) subunit, beta type 10; *Irf8* - interferon regulatory factor 8; *Icam1* - intercellular adhesion molecule 1; *Bst2* - bone marrow stromal cell antigen 2; *Trim21* - tripartite motif-containing 21; *Cd274* - cd274 antigen; *Ifit1* - interferon-induced protein with tetratricopeptide repeats 1; *Isg15* - isg15 ubiquitin-like modifier; *Ifi204* - interferon activated gene 204; *Irgm1* - immunity-related gtpase family m member 1; ***Cxcl10* - chemokine (c-x-c motif) ligand 10**; *Iigp1* - interferon inducible gtpase 1; *Irf7* - interferon regulatory factor 7; *Dtx3l* - deltex 3-like (drosophila); *Zbp1* - z-dna binding protein 1; *Sectm1b* - secreted and transmembrane 1b; *Lgals9* - lectin, galactose binding, soluble 9; *Casp1* - caspase 1; *Irf1* - interferon regulatory factor 1; *Serping1* - serine (or cysteine) peptidase inhibitor, clade g, member 1; *Ifit3* - interferon-induced protein with tetratricopeptide repeats 3; *C1ra* - complement component 1, r subcomponent a; *Gzmb* - granzyme b; *Parp9* - poly (adp-ribose) polymerase family, member 9; *Ifitm3* - interferon induced transmembrane protein 3;*Ubd* - ubiquitin d; *Nlrc5* - nlr family, card domain containing 5; *Ppbp* - pro-platelet basic protein
**GO:0006955: immune response**<br>*Samhd1* - sam domain and hd domain, 1; *Irf8* - interferon regulatory factor 8; *Icam1* - intercellular adhesion molecule 1; *Bst2* - bone marrow stromal cell antigen 2; *Trim21* - tripartite motif-containing 21; *Irgm2* - immunity-related gtpase family m member 2; ***Cxcl9* - chemokine (c-x-c motif) ligand 9**;*Cd274* - cd274 antigen; *Oas3* - 2'-5' oligoadenylate synthetase 3; *H2-T22* - histocompatibility 2, t region locus 22; *Ahcy* - s-adenosylhomocysteine hydrolase; *Ifit1* - interferon-induced protein with tetratricopeptide repeats 1; *Irgm1* - immunity-related gtpase family m member 1; ***Cxcl10* - chemokine (c-x-c motif) ligand 10**; *H2-Q4* - histocompatibility 2, q region locus 4; *Tinagl1* -

tubulointerstitial nephritis antigen-like 1; *Iigp1* - interferon inducible gtpase 1; *Irf7* - interferon regulatory factor 7; *Serpinb9* - serine (or cysteine) peptidase inhibitor, clade b, member 9; *Parp14* - poly (adp-ribose) polymerase family, member 14; *Dtx3l* - deltex 3-like (drosophila); *Zbp1* - z-dna binding protein 1; *Erap1* - endoplasmic reticulum aminopeptidase 1; *Sectm1b* - secreted and transmembrane 1b; ***Ccl1* - chemokine (c-c motif) ligand 1**; *Il18bp* - interleukin 18 binding protein; *Irf1* - interferon regulatory factor 1; *Serping1* - serine (or cysteine) peptidase inhibitor, clade g, member 1; *Trim12a* - tripartite motif-containing 12a; *Ifit3* - interferon-induced protein with tetratricopeptide repeats 3; *C1ra* - complement component 1, r subcomponent a; *Cd40lg* - cd40 ligand; *Tap2* - transporter 2, atp-binding cassette, sub-family b (mdr/tap); *Parp9* - poly (adp-ribose) polymerase family, member 9; *Tap1* - transporter 1, atp-binding cassette, sub-family b (mdr/tap); *Gzmb* - granzyme b; *Ifitm3* - interferon induced transmembrane protein 3; *Oasl2* - 2'-5' oligoadenylate synthetase-like 2; *Tnf* - tumor necrosis factor; *Nlrc5* - nlr family, card domain containing 5; *Ppbp* - pro-platelet basic protein
**GO:0051704: multi-organism process**<br>*Gbp7* - guanylate binding protein 7; *Gbp6* - guanylate binding protein 6; *Samhd1* - sam domain and hd domain, 1; *Irf8* - interferon regulatory factor 8; *Icam1* - intercellular adhesion molecule 1; *Bst2* - bone marrow stromal cell antigen 2; *Gbp3* - guanylate binding protein 3; *Mnd*a - myeloid cell nuclear differentiation antigen; *Irgm2* - immunity-related gtpase family m member 2; ***Cxcl9* - chemokine (c-x-c motif) ligand 9**; *Oas3* - 2'-5' oligoadenylate synthetase 3; *Ifit1* - interferon-induced protein with tetratricopeptide repeats 1; *Isg15* - isg15 ubiquitin-like modifier; *Ifi204* - interferon activated gene 204; *Rtp4* - receptor transporter protein 4; *Irgm1* - immunity-related gtpase family m member 1; **C*xcl10* - chemokine (c-x-c motif) ligand 10**; *Iigp1* - interferon inducible gtpase 1; *Irf7* - interferon regulatory factor 7; *Dtx3l* - deltex 3-like (drosophila); *Zbp1* - z-dna binding protein 1; *Lgals9* - lectin, galactose binding, soluble 9; *Irf1* - interferon regulatory factor 1; *Casp1* - caspase 1;*Gbp2*- guanylate binding protein 2;*Ifit3* - interferon-induced protein with tetratricopeptide repeats 3; *AA467197* - expressed sequence aa467197; *Ifi205* - interferon activated gene 205;*Parp9* - poly (adp-ribose) polymerase family, member 9; *Ifitm3* - interferon induced transmembrane protein 3; *Oasl2* - 2'-5' oligoadenylate synthetase-like 2; *Tnf* - tumor necrosis factor; *Lsm5* - lsm5 homolog, u6 small nuclear rna associated (s. cerevisiae); *Tgtp1* - t cell specific gtpase 1; *Nlrc5* - nlr family, card domain containing 5; *Psmb9* - proteasome (prosome, macropain) subunit, beta type 9 (large multifunctional peptidase 2); *Stat1* - signal transducer and activator of transcription 1; *Ppbp* - pro-platelet basic protein

1. Eden, E., et al., *GOrilla: a tool for discovery and visualization of enriched GO terms in ranked gene lists.* BMC Bioinformatics, 2009. **10**: p. 48.
