# Supplementary material for: T Cells Control Chemokine Secretion by Keratinocytes
Source: Front Immunol. 2019 Aug 9;10:1917. doi: 10.3389/fimmu.2019.01917 (PMC6696622; doi:10.3389/fimmu.2019.01917)
Supplement: Supplementary file 2 [file Data_Sheet_1.PDF]

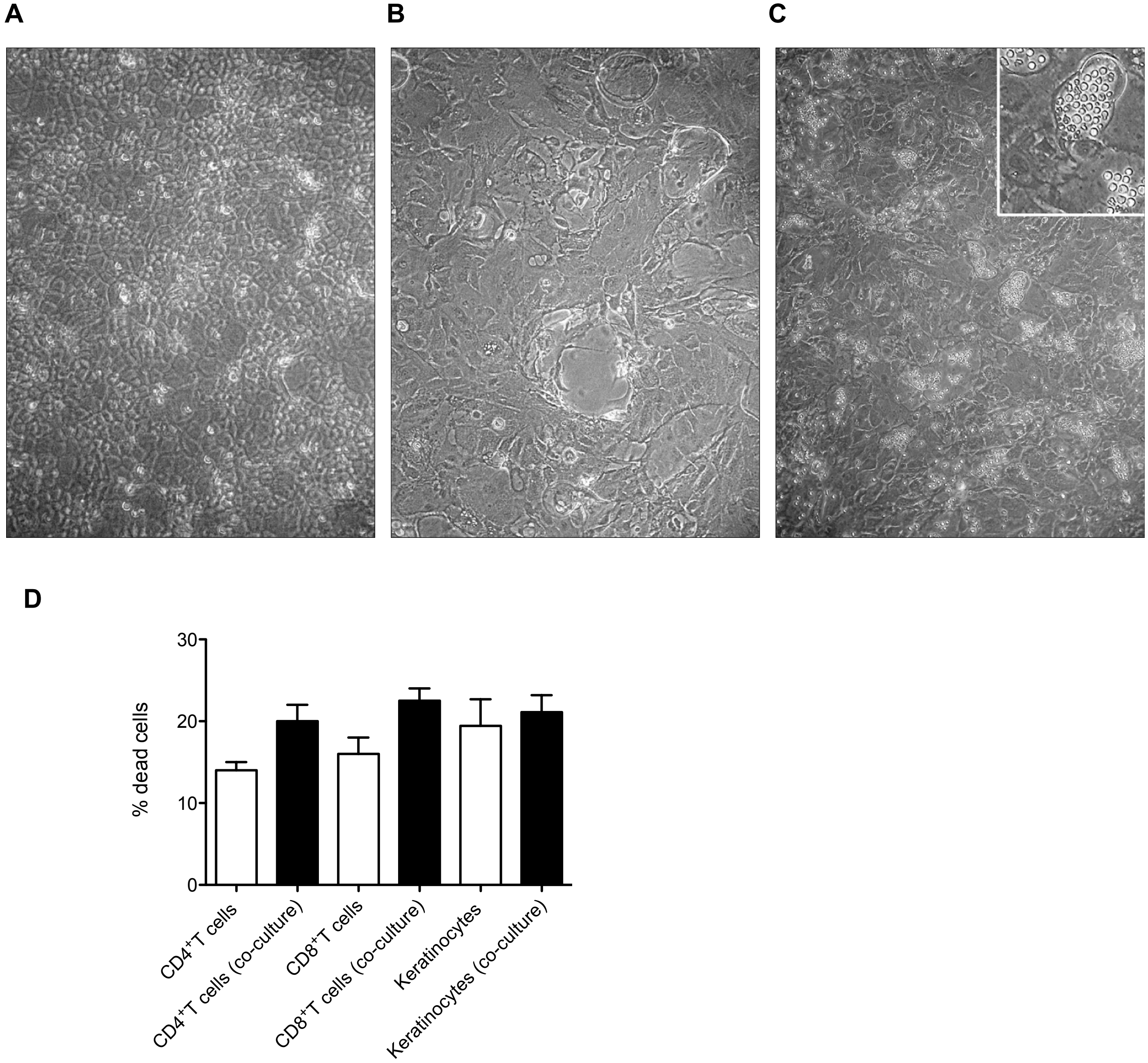

Supplementary Figure 1. Cultivation of primary keratinocytes from mouse tails. **(A)** Cobblestone-like structure of primary keratinocytes upon culture in SFM keratinocyte medium for 5 d. **(B)** Formation of holes in the lawn of primary keratinocytes upon stimulation with IL-1α and TNF-α for 1 d. **(C)** Aggregation of $CD8^+$T cells in the holes of keratinocyte cultures upon co-culture for 3 d. **(D)** Cell viability was assessed by annexin V staining before and after 1 d co-culture of T cells with keratinocytes**.**

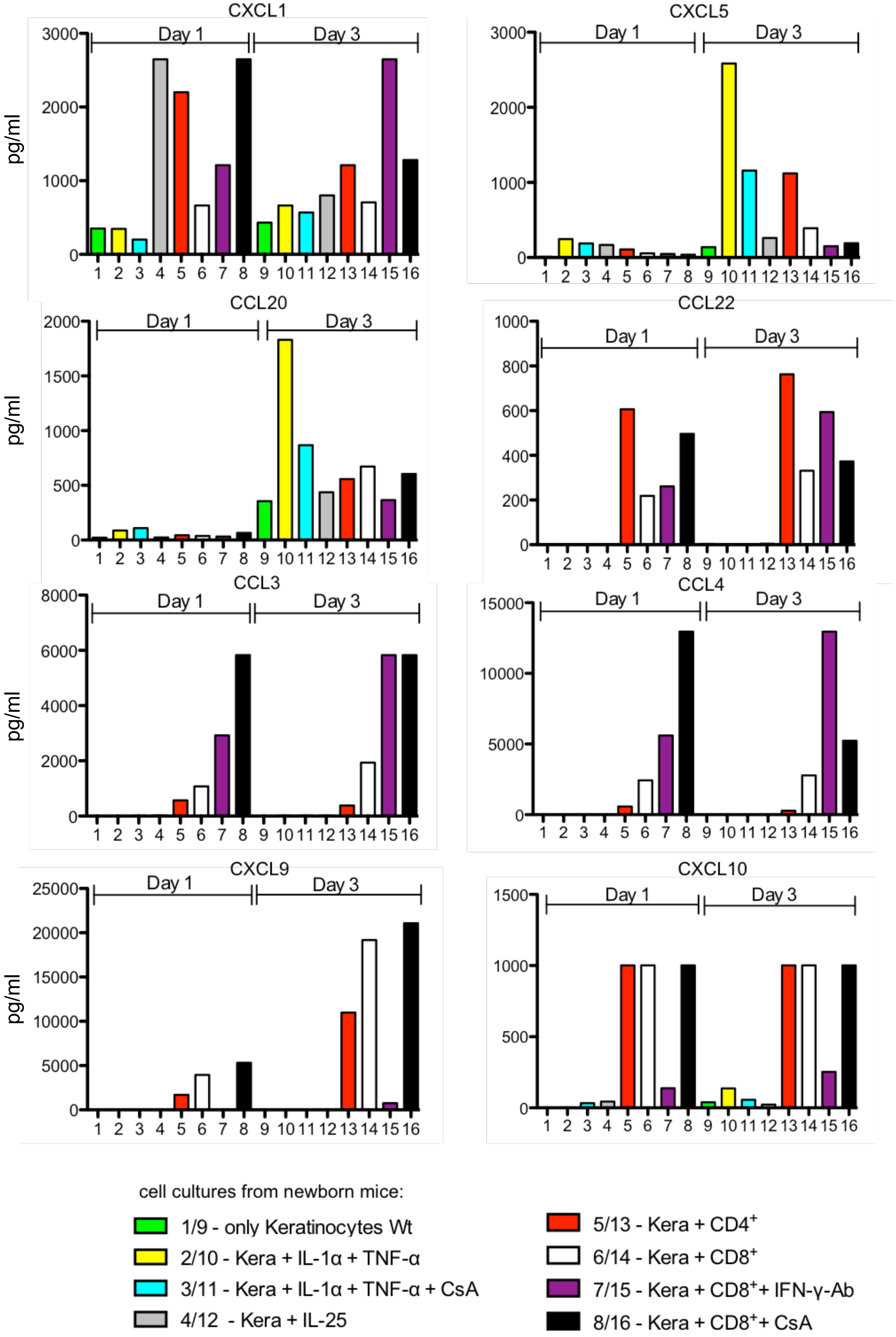

Supplementary Figure 2. Chemokine secretion of keratinocytes from newborn mice without or after co-culture with splenic murine $CD8^+$ or $CD4^+$ T cells for 1 or 3 d.

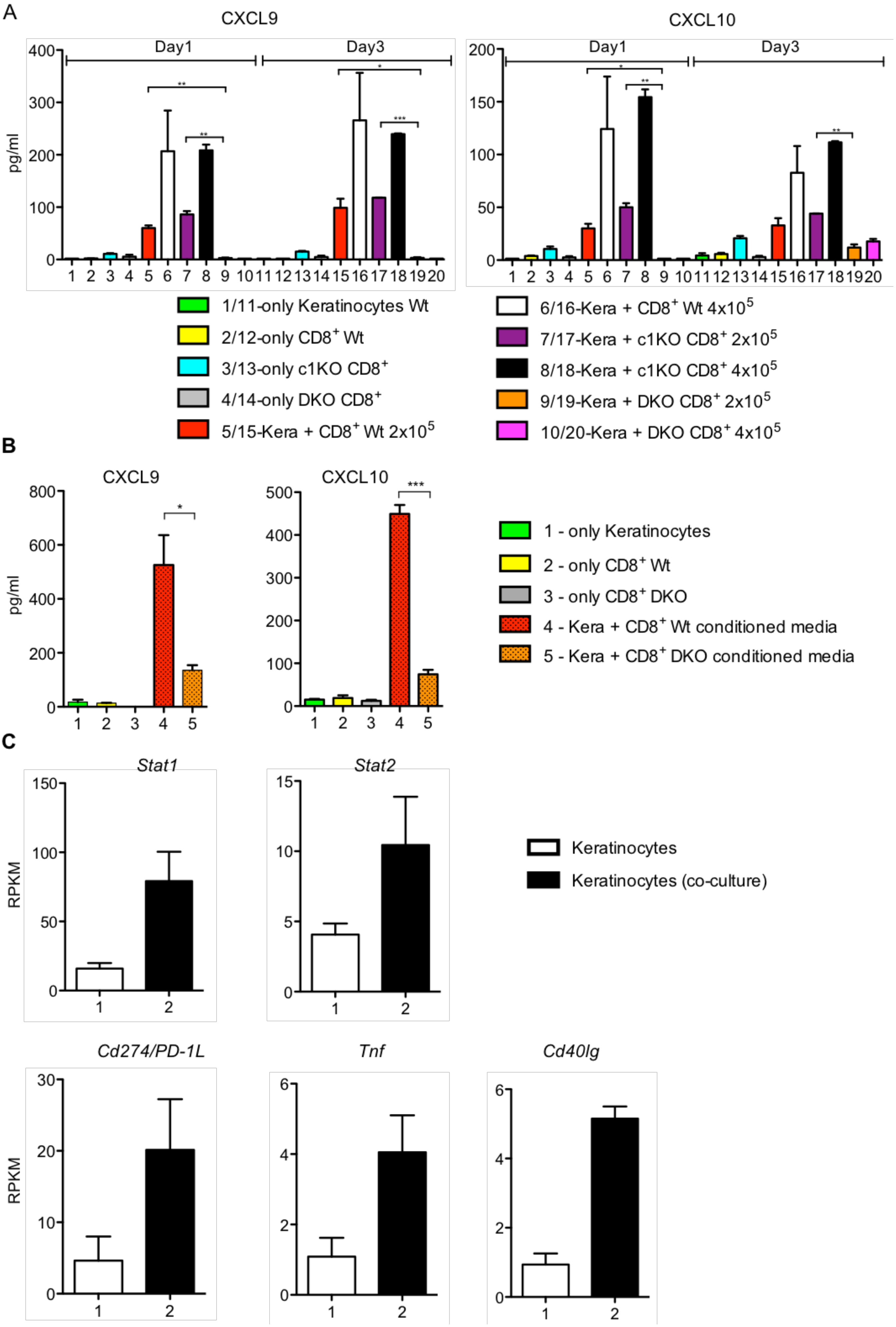
A
CXCL9
CXCL10
Day1
Day3
pg/ml
1/11-only Keratinocytes Wt
2/12-only CD8+ Wt
3/13-only c1KO CD8+
4/14-only DKO CD8+
5/15-Kera + CD8+ Wt 2x10^5
6/16-Kera + CD8+ Wt 4x10^5
7/17-Kera + c1KO CD8+ 2x10^5
8/18-Kera + c1KO CD8+ 4x10^5
9/19-Kera + DKO CD8+ 2x10^5
10/20-Kera + DKO CD8+ 4x10^5
B
CXCL9
CXCL10
pg/ml
1 - only Keratinocytes
2 - only CD8+ Wt
3 - only CD8+ DKO
4 - Kera + CD8+ Wt conditioned media
5 - Kera + CD8+ DKO conditioned media
C
Stat1
Stat2
RPKM
Keratinocytes
Keratinocytes (co-culture)
Cd274/PD-1L
Tnf
Cd40lg

Supplementary Figure 3. Induction of CXCL9 and CXCL10 secretion and of selected genes in keratinocytes upon co-culture with $CD8^+$ T cells. (**A**) Secretion of chemokines CXCL9 and CXCL10 by keratinocytes alone or in co-culture with T cells**.** Keratinocytes cultured for 7 d *in vitro*, and splenic $CD8^+$ T cells pre-activated by αCD3/CD28 Ab for 1 d were either cultured alone or together in 6-well plates for 1 or 3 d as indicated, and their chemokine secretion was measured. **(B)** Secretion of chemokines CXCL9 and CXCL10 by keratinocytes cultured in conditioned $CD8^+$ T cell medium collected after stimulation with αCD3/CD28 Ab for 1 d. (**C**) Transcriptional induction of genes encoding STAT1, STAT2, PD-1 ligand, TNF-α and CD40 ligand in keratinocytes alone or upon co-culture for 1 d with pre-activated $CD8^+$ T cells. Results of NGS assays are shown.

A

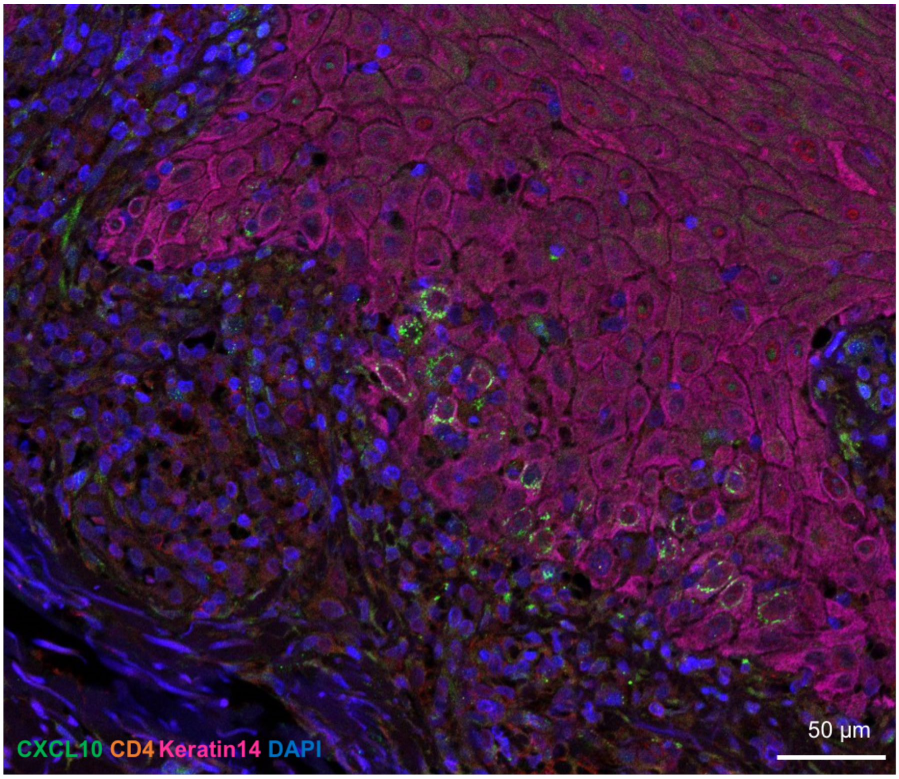

B

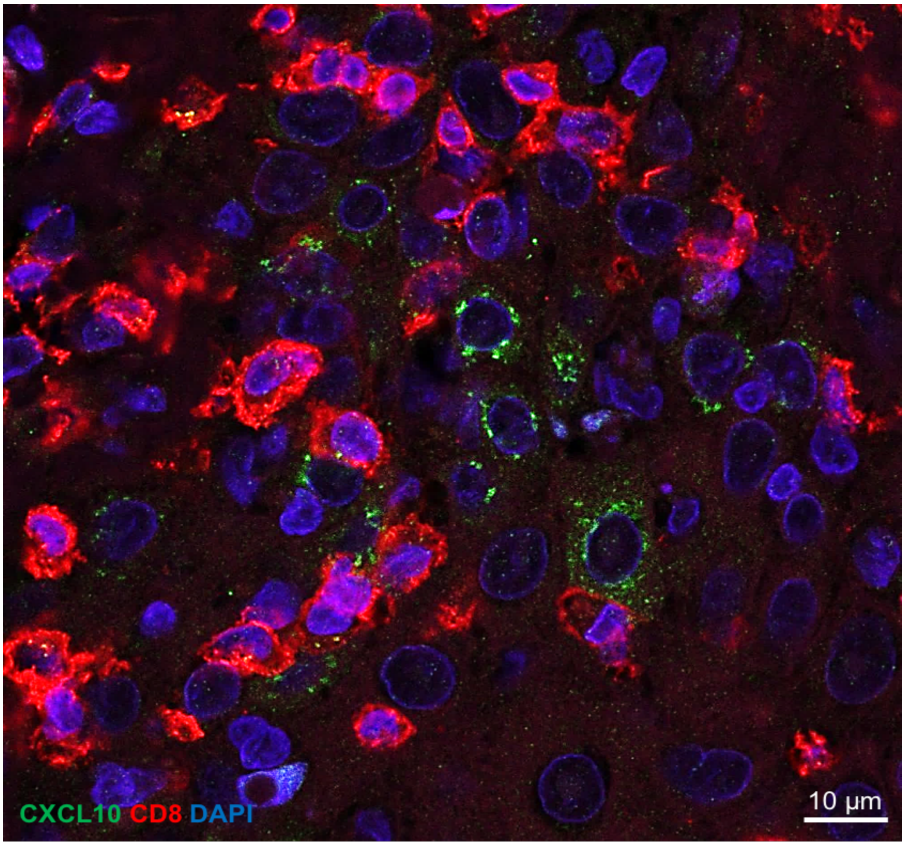

Supplementary Figure 4. Expression of CXCL10 in keratinocytes at the invasion front of T cells in skin of lichen planus patients. Lichen planus skin sections were stained with Abs directed against keratin 14, CXCL10 and CD4 **(A)** or against CXCL10 and CD8 **(B)**, and co-stained with DAPI. Confocal microscopy, 40x magnification (A) and 80x magnification(B).

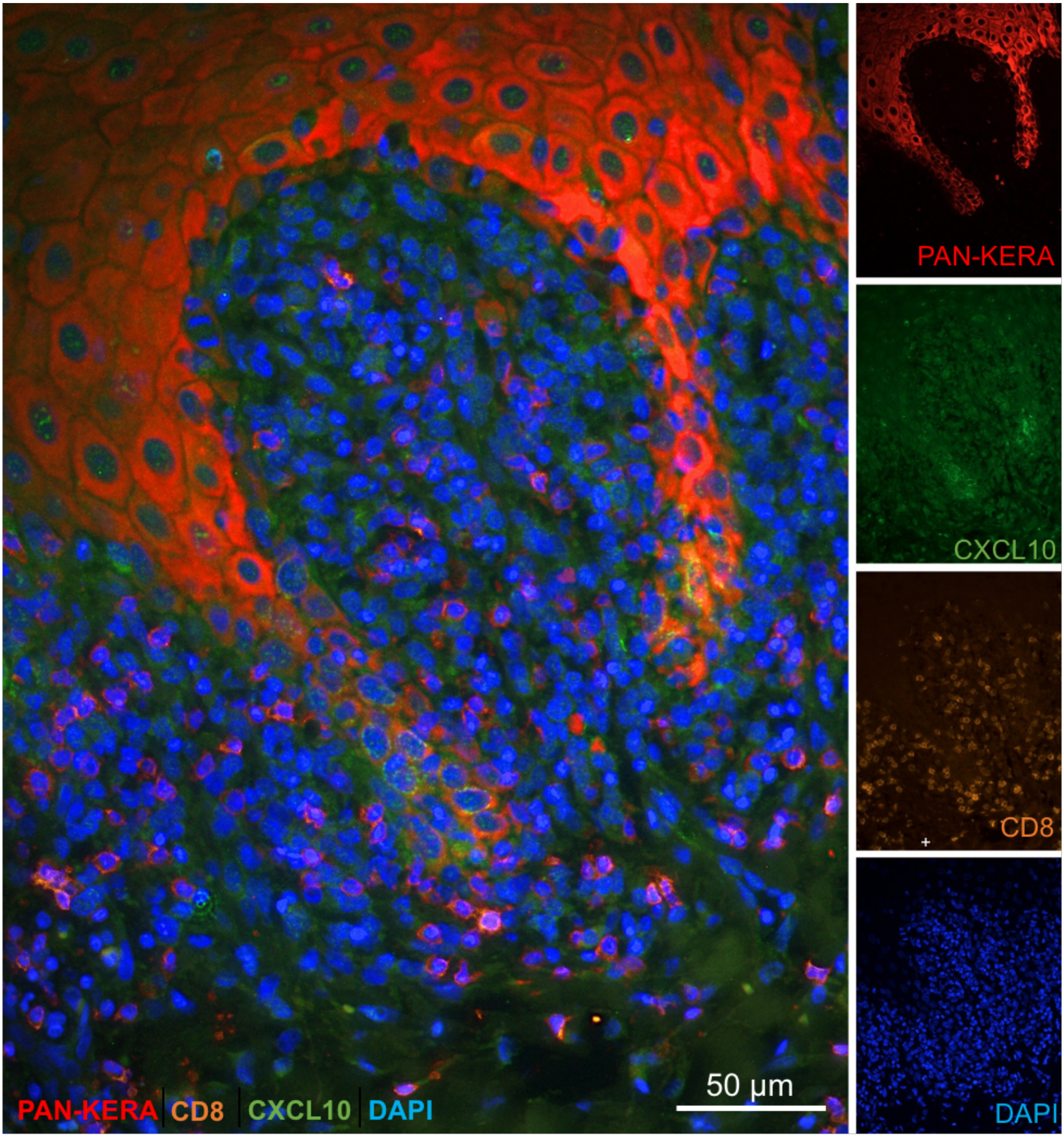

Supplementary Figure 5. Expression of CXCL10 in keratinocytes at the invasion front to T cells in lichen planus. Skin sections were stained with Abs directed against pan-keratin, CXCL10 and CD8 and co-stained with DAPI. Immunofluorescence microscopy, 40x magnification.

A

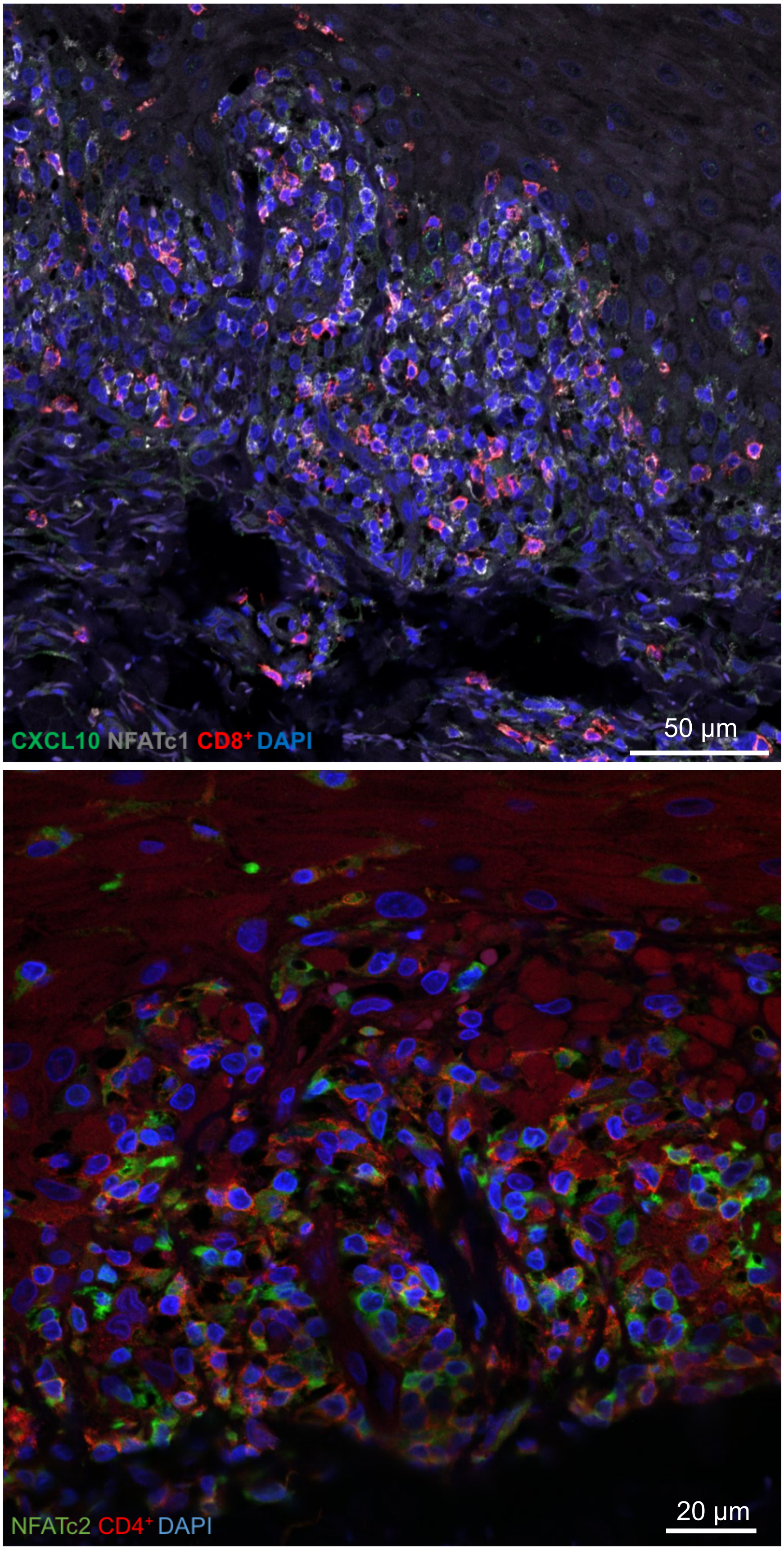

B

Supplementary Figure 6. Cytoplasmic expression of NFAT factors NFATc1 and NFATc2 in invading T cells in the skin of lichen planus patients. Skin sections were stained with Abs directed against CXCL10, NFATc1 and CD8 **(A)** or against NFATc2 and CD4 **(B)** and co-stained with DAPI. Confocal microscopy, 20x (A) and 40x (B) magnification.
